# Development of RAP Tag, a Novel Tagging System for Protein Detection and Purification

**DOI:** 10.1089/mab.2016.0052

**Published:** 2017-04-01

**Authors:** Yuki Fujii, Mika K. Kaneko, Satoshi Ogasawara, Shinji Yamada, Miyuki Yanaka, Takuro Nakamura, Noriko Saidoh, Kanae Yoshida, Ryusuke Honma, Yukinari Kato

**Affiliations:** ^1^ Department of Regional Innovation, Tohoku University Graduate School of Medicine, Sendai, Japan.; ^2^ New Industry Creation Hatchery Center, Tohoku University, Sendai, Japan.

**Keywords:** monoclonal antibody, RAP tag, affinity tag, protein purification, podoplanin

## Abstract

Affinity tag systems, possessing high affinity and specificity, are useful for protein detection and purification. The most suitable tag for a particular purpose should be selected from many available affinity tag systems. In this study, we developed a novel affinity tag called the “RAP tag” system, which comprises a mouse antirat podoplanin monoclonal antibody (clone PMab-2) and the RAP tag (DMVNPGLEDRIE). This system is useful not only for protein detection in Western blotting, flow cytometry, and sandwich enzyme-linked immunosorbent assay, but also for protein purification.

## Introduction

Affinity tag systems, which are useful for protein purification and detection, are classified into “peptide tags” and “protein tags.” Protein tags, including glutathione-S-transferase tag,^(1)^ maltose-binding protein tag,^(2)^ Fc tag of immunoglobulin,^(3)^ and green fluorescent protein tag,^(4)^ have numerous advantages because they are useful for protein expression in the soluble fraction or are easily detected using monoclonal antibodies (mAbs). In contrast, protein tags sometimes affect the character of the target proteins if they are large in size (>25 kDa); therefore, their removal is necessary before any protein analysis. Peptide tags are less likely to affect the structure and function of target proteins because of their small size (typically 1–2 kDa); therefore, it is not always necessary to remove the tag portion for protein analysis.

The most appropriate peptide tag system can be selected from many available peptide tags, such as FLAG tag,^(5)^ TARGET tag,^(6)^ PA tag,^(7–16)^ and MAP tag,^(17–20)^ among many others.^(21–23)^ These systems sometimes have disadvantages, such as low specificity, low affinity, or difficulty in achieving protein elution. Therefore, we need to develop further affinity tag systems to overcome these disadvantages of established affinity tag systems.

We previously developed a mouse mAb (clone PMab-2) against the platelet aggregation-stimulating domain of rat podoplanin.^(24)^ Podoplanin is a type I transmembrane protein, which is highly expressed in many normal cells and cancer cells, and is involved in tumor-induced platelet aggregation by binding to CLEC-2 on platelets.^(3,25–33)^ Because PMab-2 possesses high affinity and specificity against rat podoplanin,^(24)^ it was expected to be useful as an antitag antibody. Herein, we developed a novel affinity tag system, the “RAP tag” system, using PMab-2 mAb.

## Results and Discussion

**FIG. 1.**
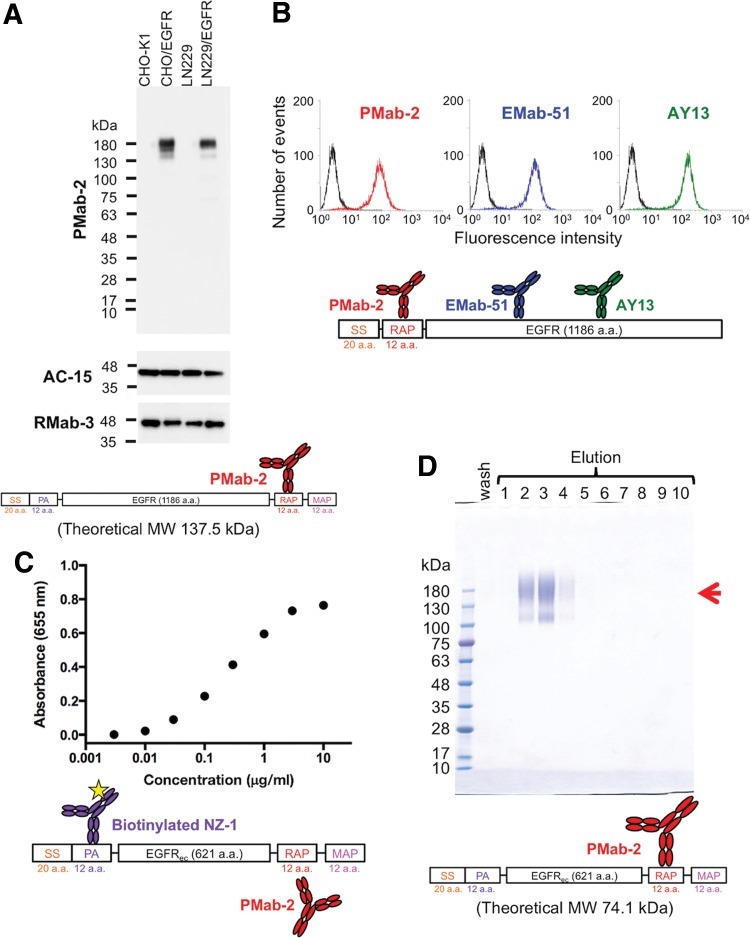
Detection and purification of RAP-tagged EGFR by the RAP tag system. **(A)** Western blot analysis of RAP-tagged EGFR using anti-RAP tag mAb, PMab-2. Total cell lysates (CHO-K1, CHO/EGFR, LN229, and LN229/EGFR) were electrophoresed under reducing conditions using 5%–20% SDS-PAGE gel and transferred to a membrane. The membrane containing the same amount of lysate was immunoblotted using 1 μg/mL PMab-2 (anti-RAP tag), AC-15 (anti-β-actin), or RMab-3 (anti-IDH1) for 30 minutes, and incubated with a peroxidase-conjugated secondary antibody specific for mouse IgG. **(B)** Flow cytometric analysis of RAP-tagged membrane protein. Two antihuman EGFR mAbs (clones: EMab-51 and AY13) were used in this study. EMab-51 (IgG_1_, κ) was established in our laboratory. AY13 (IgG_1_, κ) was purchased from BioLegend (San Diego, CA). CHO-K1 cells and CHO/RAP-EGFR were treated with 1 μg/mL PMab-2, EMab-51, or AY13 for 30 minutes at 4°C, followed by 1:1000 diluted Oregon Green 488 goat antimouse IgG (Thermo Fisher Scientific, Inc., Waltham, MA). Fluorescence data were collected using a Cell Analyzer EC800 (Sony Corp., Tokyo, Japan). **(C)** Sandwich ELISA of RAP-tagged protein. PMab-2 was immobilized at a concentration of 10 μg/mL for 30 minutes. After blocking with 1% bovine serum albumin in 0.05% Tween 20/phosphate-buffered saline (pH 7.4), recombinant PA-EGFR_ec_-RAP-MAP was added at a concentration from 3 ng/mL to 10 μg/mL and was incubated overnight. After washing, the plates were incubated with 0.5 μg/mL biotinylated NZ-1 (anti-PA tag), followed by 1:5000 diluted peroxidase-conjugated streptavidin (GE Healthcare Bio-Sciences, Pittsburgh, PA). The enzymatic reaction was performed using 1-Step Ultra TMB-ELISA (Thermo Fisher Scientific, Inc.). The optical density was measured at 655 nm using an iMark microplate reader (Bio-Rad Laboratories, Inc., Berkeley, CA). Data are means of four replicates ± SEM. **(D)** Purification of soluble ectodomain fragment of EGFR (EGFR_ec_). LN229/EGFR_ec_ was cultured and 1 L of culture supernatant was harvested. The filtered supernatant was passed through PMab-2-Sepharose (4 mL bed volume), and the same process was repeated three times. The beads were then washed with 80 mL of Tris-buffered saline (TBS; pH 7.5) and eluted with 0.1 mg/mL epitope peptide in a step-wise manner (4 mL × 10). Ten microliters of the 5th of 5 washes in TBS (wash) and 10 peptide-eluted fractions (lanes 1–10) during the column chromatography were subjected to 5%–20% SDS-PAGE under reducing conditions and were stained with Coomassie brilliant blue. Arrow: EGFR_ec_. ec, ectodomain; EGFR, epidermal growth factor receptor; ELISA, enzyme-linked immunosorbent assay; mAb, monoclonal antibody; SDS-PAGE, sodium dodecyl sulfate polyacrylamide gel electrophoresis; SS, signal sequence.

We first investigated the binding affinity between PMab-2 mAb and RAP tag (DMVNPGLEDRIE) using the BIAcore X100 system. Curve fitting showed the affinity for PMab-2–RAP tag interaction: *k_a_* = 2.0 × 10^5^ M^−1^ s^−1^, *k_d_* = 2.0 × 10^−3^ s^−1^, and *K*_D_ = 9.7 × 10^−9^ M, indicating that PMab-2 showed moderate affinity toward the RAP tag. Next, we examined whether the RAP tag system is useful for several protein detection systems. As depicted in Figure 1A, PMab-2 showed a strong single band corresponding to the molecular weight of human epidermal growth factor receptor (EGFR) with RAP tag (∼170 kDa) in Western blot analysis. Transfectants of EGFR were established previously.^(17)^ PMab-2 did not show any nonspecific bands in CHO-K1 and LN229 cells, indicating that PMab-2 is very specific to the RAP tag. PMab-2 reacted with the EGFR with the N-terminal RAP tag, which was expressed in CHO-K1 cells in flow cytometry (Fig. 1B, left). As a positive control, antihuman EGFR mAbs (EMab-51 and AY13) recognized CHO-K1/EGFR (Fig. 1B, middle and right). We further investigated whether the RAP tag system is useful in sandwich enzyme-linked immunosorbent assay (ELISA). PMab-2 was immobilized, and the soluble ectodomain fragment of EGFR (EGFR_ec_) with RAP tag was added at a concentration from 3 ng/mL to 10 μg/mL and detected by biotinylated NZ-1. As shown in Figure 1C, EGFR_ec_ was detected in a dose-dependent manner. These results indicate that the RAP tag system is useful for Western blotting, flow cytometry, and sandwich ELISA.

Next, we purified three different RAP-tagged proteins using the RAP tag system. The EGFR_ec_ and the soluble ectodomain fragment of human epidermal growth factor receptor 2 (HER2_ec_) were expressed in LN229 and were purified from the culture supernatant (Fig. 1D and Supplementary Fig. S1A). The fragment of α-thalassemia/mental-retardation-syndrome-X-linked (ATRXepi; ∼25 kDa) was expressed in *Escherichia coli* and purified from the soluble fraction of the bacterial lysate (Supplementary Fig. S1B). All RAP-tagged proteins were captured onto PMab-2-Sepharose and eluted from the resin by a solution containing 0.1 mg/mL free epitope peptide (GDDMVNPGLEDRIE). These proteins were efficiently eluted using the RAP tag peptide because of the high dissociation constant for PMab-2–RAP tag interaction. EGFR_ec_ and HER2_ec_ were eluted in elution fractions 2–4 (EGFR_ec_) or 2–3 (HER2_ec_) at a high concentration (Fig. 1D and Supplementary Fig. S1A). EGFR_ec_ and HER2_ec_ are highly glycosylated^(34,35)^; therefore, they were electrophoresed at a “heavier” position compared with that suggested by the theoretical molecular weight. ATRXepi was eluted in elution fractions 2–10 (Supplementary Fig. S1B). These purified proteins showed high purity and were ready for use in downstream experiments without further purification, indicating that the RAP tag system is a powerful protein purification tool.

In conclusion, we successfully developed a novel affinity tagging system, “RAP tag,” by employing a unique mAb PMab-2 against rat podoplanin. The RAP tag system could be advantageous for protein purification and detection in the field of protein science.

## Supplementary Material

Supplemental data
