# Supplementary material for: Development of RAP Tag, a Novel Tagging System for Protein Detection and Purification
Source: Monoclon Antib Immunodiagn Immunother. 2017 Apr 1;36(2):68–71. doi: 10.1089/mab.2016.0052 (PMC5404252; doi:10.1089/mab.2016.0052)
Supplement: Supplemental data [file Supp_Figure1.pdf]

Supplementary Data

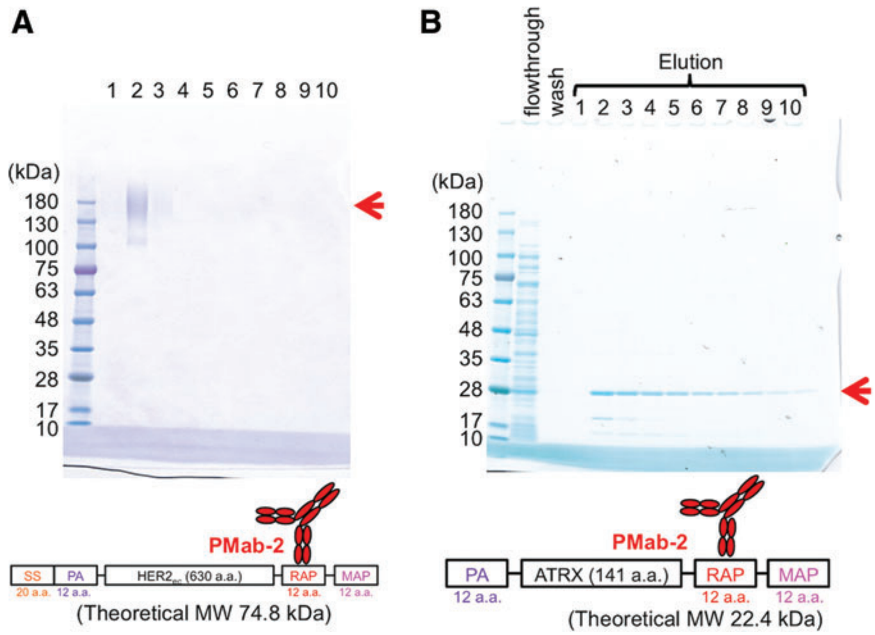

SUPPLEMENTARY FIG. S1. Purification of RAP-tagged proteins by the RAP tag system. **(A)** Purification of soluble ectodomain fragment of HER2 ($HER2_{ec}$). For the purification of $HER2_{ec}$, LN229/$HER2_{ec}$ was cultivated and 1 L of culture supernatant was harvested. The filtered supernatant was passed through PMab-2-Sepharose (4 mL bed volume), and the same process was repeated three times. The beads were then washed with 80 mL of TBS (pH 7.5) and eluted with 0.1 mg/mL epitope peptide (GDDMVNPGLEDRIE) in a step-wise manner (4 mL × 10). Five microliters of ten peptide-eluted fractions (lanes 1–10) during the column chromatography were subjected to 5%–20% SDS-PAGE under reducing conditions and stained with CBB. Arrow: $HER2_{ec}$. **(B)** Purification of ATRXepi from the transformed *Escherichia coli*. The recombinant ATRXepi (PA-ATRXepi-RAP-MAP) was expressed in transformed *E. coli* BL21 (DE3), which was cultured for ~3 hours at 37°C in the presence of 1-mM IPTG. The soluble fraction was mixed with PMab-2-Sepharose (1 mL bed volume) and incubated at 4°C for 3 hours under gentle agitation. The beads were washed with 20 mL of TBS. The bound protein was eluted with 0.1 mg/mL epitope peptide. The elution was conducted at room temperature in a step-wise manner (1 mL × 10). Five microliters of the soluble fraction after PMab-2-Sepharose capture (flowthrough), the 5th of 5 washes in TBS (wash), and 10 peptide-eluted fractions (lanes 1–10) during the column chromatography were subjected to 5%–20% SDS-PAGE under reducing conditions and stained with CBB. Arrow: ATRX. ATRX, α-thalassemia/mental-retardation-syndrome-X-linked; CBB, Coomassie brilliant blue; ec, ectodomain; HER2, human epidermal growth factor receptor 2; SDS-PAGE, sodium dodecyl sulfate polyacrylamide gel electrophoresis; TBS, tris-buffered saline.
